# Ultrasound features and thyroid function of patients with papillary thyroid carcinoma for predicting metastatic cervical lymph nodes with atypical ultrasound features

**DOI:** 10.3389/fonc.2025.1628205

**Published:** 2025-08-08

**Authors:** Shuting Li, Wen Tang, Mengjuan Feng, Zonghui Zhang, Zhongping Tang, Yuanyuan Yue

**Affiliations:** ^1^ Department of Ultrasound, Chengdu Integrated TCM and Western Medicine Hospital, Chengdu, Sichuan, China; ^2^ Department of Pathology, Chengdu Integrated TCM and Western Medicine Hospital, Chengdu, Sichuan, China

**Keywords:** ultrasonography, papillary thyroid carcinoma, thyroglobulin, lymphatic metastasis, nomogram, risk factor, predictive model

## Abstract

**Objective:**

To develop a nomogram based on ultrasound features and preoperative serum thyroid function of patients with primary papillary thyroid carcinoma (PTC) to quantify the probability of atypical metastatic cervical lymph nodes.

**Methods:**

A retrospective study involving 316 patients diagnosed with PTC at Chengdu Integrated TCM & Western Medicine Hospital from January 2023 to December 2024 was conducted. Patients with typical ultrasound features of metastatic cervical lymph nodes or incomplete data were excluded, and 158 PTC patients with atypical ultrasound features were included in the study. The patients were divided into two groups based on the presence of cervical lymph node metastasis in the postoperative pathologic findings. The thyroid function and ultrasound data of the two groups were analyzed to identify independent risk factors for metastatic cervical lymph nodes with atypical ultrasound features. A nomogram prediction model was established and evaluated for discrimination and calibration via receiver operating characteristic (ROC) curves, calibration curves, and 5-fold cross-validation.

**Results:**

Of the 158 patients, 59 were assigned to the metastatic group, and 99 were assigned to the nonmetastatic group. Multivariate analysis revealed the following independent risk factors for metastatic cervical lymph nodes with atypical ultrasound features: age ≤ 45 years (OR=2.898, 95% CI=1.294-6.810), male sex (OR=3.224, 95% CI=1.468-7.333), contact with capsule (OR=7.346, 95% CI=2.448-27.049), internal blood flow (grade II-III, OR=4.915, 95% CI=1.626-15.882), and TGAb positivity (OR=5.173, 95% CI=2.026-14.355). Based on these factors, a nomogram model was developed, which demonstrated an AUC of 0.805, a sensitivity of 72.88%, a specificity of 76.77%, and an accuracy of 75.32%.

**Conclusion:**

The nomogram, which is based on age, sex, the distance between the nodule and the adjacent capsule, internal blood flow, and TGAb levels, has a strong ability to predict cervical lymph node metastasis in PTC patients with atypical ultrasound features. This model may assist in reducing the incidence of misdiagnoses of metastatic lymph nodes by providing imaging and laboratory data to facilitate clinical decision-making.

## Introduction

Thyroid cancer has an incidence rate of up to 5% and is the most common endocrine malignancy, with an increasing incidence in recent years (1, 2). Among thyroid cancers, papillary thyroid carcinoma (PTC) is the predominant type, accounting for 85–95% of all cases (3, 4). Although PTC generally has a favorable prognosis, studies have shown that 30–80% of patients with PTC develop cervical lymph node metastasis (LNM), which is strongly associated with tumor recurrence (5). The detection of cervical lymph node metastasis in postoperative pathology may necessitate secondary surgery (6), complicating the procedure and increasing the risk of permanent hypoparathyroidism and recurrent laryngeal nerve injury. Therefore, it is crucial to identify preoperative risk factors and accurately predict the possibility of lymph node metastasis.

Preoperative ultrasound of the thyroid and cervical lymph nodes is the most common method used to assess LNM in PTC patients. It also serves as a key tool in determining the extent of cervical lymph node dissection and holds significant clinical value. Typical ultrasound features of metastatic cervical lymph nodes, such as punctate high echoes, hyperechogenicity, or cystic changes, exhibit high diagnostic specificity (up to 88.6%) (7). However, the sensitivity of preoperative ultrasound for diagnosing cervical lymph node metastasis in patients with PTC is relatively low (10–35.3%) (8, 9). This low sensitivity is due primarily to the small size of many atypical metastatic lymph nodes and the absence of specific ultrasound features.

Thyroid function tests serve as objective biomarkers of thyroid activity and can be used both preoperatively to assess thyroid function and postoperatively to evaluate recovery. For example, elevated anti-thyroglobulin antibodies (TGAb) have been shown to predict outcomes in patients with PTC (10). The nomogram model is an objective statistical tool that helps mitigate subjective bias in the diagnostic process. However, few studies have incorporated thyroid function test indicators into predictive models. This study aims to identify predictive factors for atypical metastatic lymph node ultrasound features by retrospectively analyzing the sonographic characteristics of primary PTC lesions alongside thyroid function test indicators. The ultimate goal is to develop a nomogram prediction model to assist in surgical decision-making.

## Methods

### Study population and design

This retrospective study was approved by the Medical Ethics Review Committee of the Chengdu Integrated TCM & Western Medicine Hospital (Approval No. 2024 YNYJ No.028). Due to the retrospective nature of the study, the Medical Ethics Review Committee of the Chengdu Integrated TCM & Western Medicine Hospital waived the need to obtain informed consent. This study adheres to the Strengthening and Reporting of Observational Studies in Epidemiology (STROBE) Statement and Transparent Reporting of a Multivariable Prediction Model for Individual Prognosis or Diagnosis (TRIPOD) guidance for prediction model development and validation. The study adhered to the principles of the Declaration of Helsinki and its subsequent revisions. We retrospectively analyzed the ultrasound images, thyroid function test results, and clinical characteristics of 316 patients diagnosed and treated at the Chengdu Integrated TCM & Western Medicine Hospital between January 2023 and December 2024.

The inclusion criteria for patients were as follows: pathologically confirmed PTC; a single thyroid nodule; the first thyroid surgery with cervical lymph node dissection; metastatic lymph node with atypical ultrasound features were met, definition of the atypical metastatic lymph node is an abnormally structured lymph node that does not meet the criteria for a typical metastatic lymph node. The atypical metastatic lymph node ultrasound features include lymph nodes that are round or roundish, loss or eccentricity of the lymph node hilum, or poor demarcation of the corticomedullary stroma, irregular or blurred borders (**Table 1**). The exclusion criteria were as follows: combined with other types of thyroid cancer (e.g., medullary and follicular thyroid carcinoma); uncertain pathological diagnosis of thyroid nodules; combined with other malignant tumors (e.g., lymphoma, liver cancer, or breast cancer); and incomplete clinical and ultrasound information.

**Table 1:** Atypical and typical ultrasound features of metastatic cervical lymph nodes.

Variable	Atypical ultrasound features of metastatic cervical lymph nodes	Typical ultrasound features of metastatic cervical lymph nodes
1	Lymph nodes are round or roundish	punctate high echoes
2	Loss or eccentricity of the lymph node hilum or poor demarcation of the corticomedullary stroma	hyperechogenicity
3	Irregular or blurred borders	cystic changes

**Figure 1:**
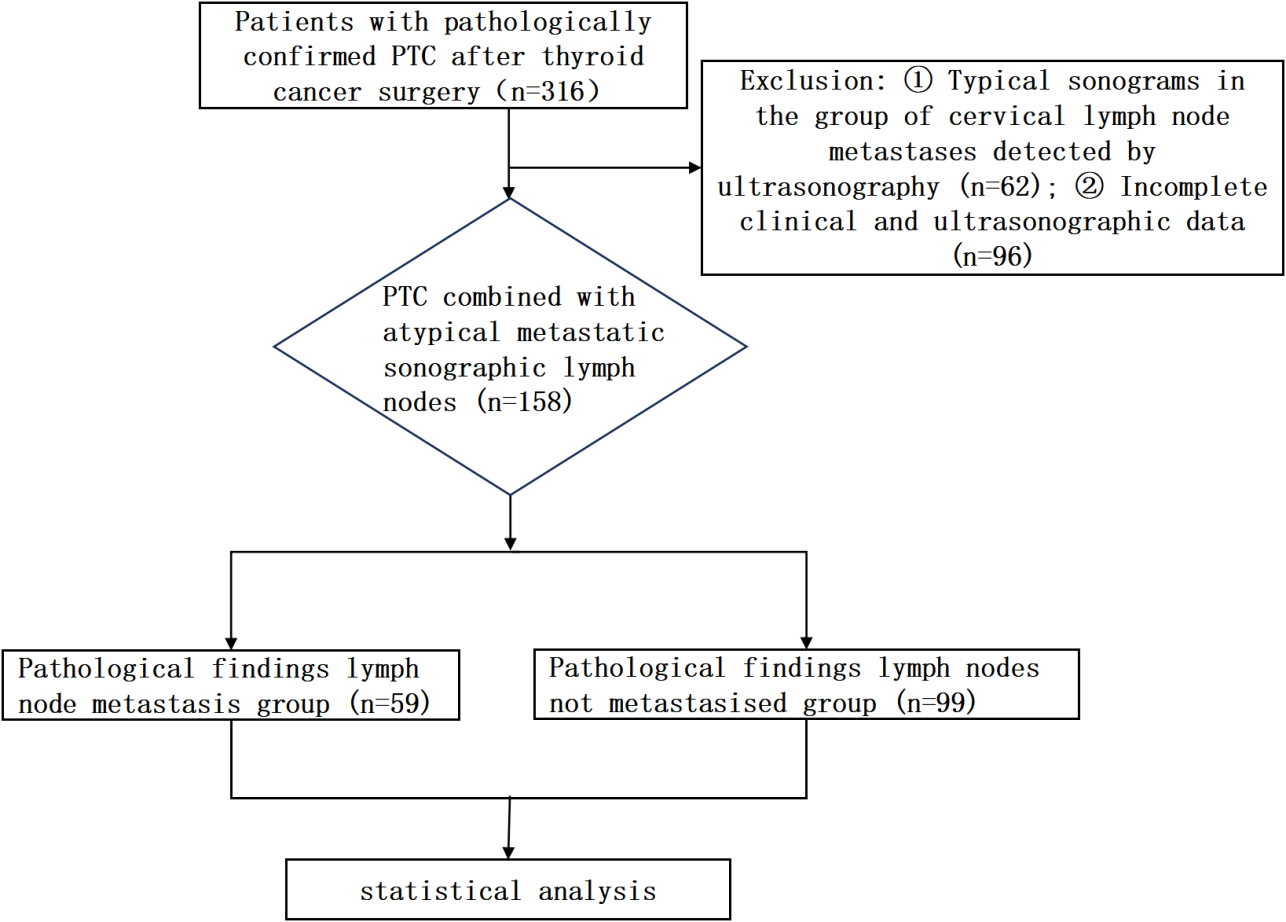
The flow chart of the study.

The patient study flowchart is shown in **Figure 1**. A total of 158 PTC patients with atypical cervical lymph nodes were included in the final analysis. Among these, 60 were male (mean age 40.33 ± 13.53 years), and 98 were female (mean age 43.44 ± 11.64 years). According to the 2017 TNM staging system of the American Joint Committee on Cancer (AJCC) (11), all patients enrolled were identified as T_0-3_N_0-1_M_0_. Preoperative thyroid nodules were determined to be PTC by puncture biopsy, and preoperative imaging revealed an abnormally structured lymph node, which would be punctured. According to the 8th edition of the AJCC guidelines, thyroid lobectomy was chosen for nodules less than 2 cm, single, and without extrathyroidal extension, and total thyroidectomy was chosen for nodules greater than 2 cm, multiple, or the presence of extrathyroidal extension. At the time of surgery, the central lymph nodes will be cleared and sent for intraoperative freezing, and the surgery will be terminated if the result of frozen pathology is negative; if the result of frozen pathology suggests the presence of lymph node metastasis, then the scope will be expanded to clear the lymph nodes in the neck (9, 11). Patients were classified into a metastatic group (n = 59) and a nonmetastatic group (n = 99) on the basis of their postoperative pathological results.

### Instruments and methods

**Figure 2:**
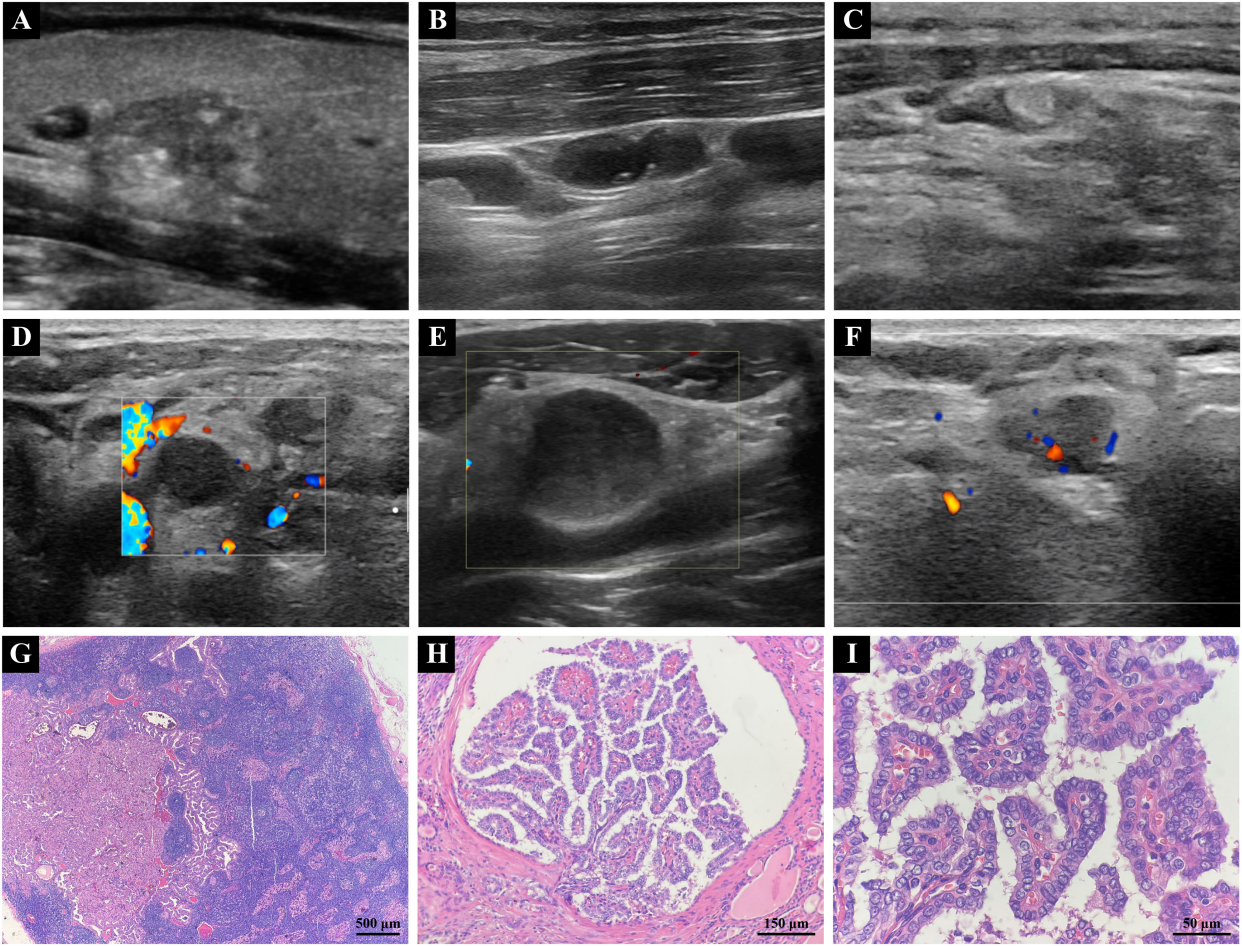
**(A)** Two-dimensional grayscale ultrasound image of PTC. **(B)** Typical ultrasonographic features of metastatic lymph node in PTC, showing cystic changes and punctate echogenic foci. **(C)** Typical ultrasonographic features of metastatic lymph nodes in PTC, demonstrating hyperechoic masses. **(D)** Atypical ultrasonographic features of metastatic lymph node in PTC, Lymph nodes are round or roundish. **(E)** Atypical ultrasonographic features of metastatic lymph node in PTC, Loss or eccentricity of the lymph node hilum or poor demarcation of the corticomedullary stroma. **(F)** Atypical ultrasonographic features of metastatic lymph node in PTC, Irregular or blurred borders. **(G)** Metastasis of PTC tumor identified within a cervical lymph node (4X). **(H)** PTC tumor exhibiting a branching papillary structure with invasive growth within thyroid tissue (20X). **(I)** PTC tumor cells showing enlargement, crowding, and overlapping, with ground-glass nuclei and visible nuclear grooves (60X). (PTC, papillary thyroid carcinoma).

Preoperative ultrasound examinations were performed utilizing the Aixplorer ultrasound scanning system (SuperSonic Imagine, France) equipped with a 4-15MHz linear array transducer. During the ultrasound examination procedure, patients were placed in a supine position with a small pillow positioned beneath the head to facilitate a slight backward tilt. Transducer settings, including frequency, depth, focal zone, gain, magnification, and dynamic range, were optimized for each patient to ensure optimal image quality. Comprehensive scanning revealed the entirety of the thyroid gland as well as the cervical lymph nodes (**Figure 2**). The maximum longitudinal and transverse views of each lesion were documented through grayscale ultrasound and color Doppler imaging. Two experienced physicians, each possessing over 10 years of expertise in thyroid ultrasound diagnostics, independently conducted a blinded retrospective analysis of the collected images. Any diagnostic discrepancies were subsequently resolved through consensus-based discussion.

The clinical characteristics and thyroid function laboratory indicators recorded for PTC patients included age, sex, body mass index (BMI), the presence of Hashimoto’s thyroiditis confirmed by postoperative pathological examination, and the measurements of preoperative serum thyroid function and thyroid autoantibodies conducted by radioimmunoassay. These measurements included free triiodothyronine (FT3) (reference: 3.53-7.37 pmol/L), free thyroxine (FT4) (reference: 7.90-16.01 pmol/L), thyroid stimulating hormone (TSH) (reference: 0.560-5.910 mIU/L), anti-thyroid peroxidase antibodies (TPO-AB) (reference: < 9.00 IU/ml), anti-thyroglobulin antibodies (TGAb) (reference: < 4.00 IU/ml; TGAb < 4.00 IU/ml was defined as negative, TGAb ≥ 4.00 IU/ml was defined as positive), and BRAF V600E mutation. PTC primary lesions were tested for the BRAF V600E mutation by PCR.

The primary ultrasound characteristics recorded for PTC nodules included lobulation, anatomical location, maximum diameter, margin, shape (the anteroposterior dimension divided by its transverse dimension, A/T), echogenicity, homogeneity of internal echoes, distance between the nodule and the adjacent capsule (the relationship between the nodule and adjacent capsule was classified into two categories as follows: (1) contact with capsule: ≤ 2 mm and protruding outside the thyroid capsule; (2) noncontact with capsule: > 2 mm), echogenic foci (categorized as microcalcifications, which are defined as calcifications <1mm along the longest axis; nonmicrocalcifications, including coarse calcifications, or absence of calcifications), and internal blood flow was classified following the Adler criterion from grade 0 to grade III and evaluated by color Doppler flow imaging (CDFI) (12).

### Statistical methods

The data were analyzed using SPSS version 25.0 (IBM Corp, Armonk, NY, USA). Continuous variables with a normal distribution, such as BMI, are expressed as the mean ± standard deviation (SD). Continuous variables that did not follow a normal distribution, such as the maximum nodule diameter, are expressed as the median (interquartile range, IQR), and comparisons between groups were made using the nonparametric Wilcoxon rank-sum test. Categorical variables are presented as frequencies (percentages), with comparisons performed using Pearson’s chi-square test or Fisher’s exact test, as appropriate. Univariate and multivariate logistic regression analyses were conducted to identify independent risk factors associated with atypical metastatic cervical lymph nodes based on ultrasound imaging characteristics and serum thyroid function. A nomogram was constructed based on these results using R version 4.3.1 software (R Foundation for Statistical Computing, Vienna, Austria). The dataset of 158 cases was first divided into 8:2. 80% of the dataset was subjected to predictive modeling and 5-fold cross-validation. The remaining 20% was subjected to predictive model testing. The performance of the nomogram was evaluated by plotting the receiver operating characteristic (ROC) curve, and the area under the curve (AUC) was calculated. A calibration curve was also generated to assess the model’s fit. A two-tailed P value of <0.05 was considered to indicate statistical significance.

## Results

### General information

A total of 158 PTC patients with atypical ultrasound findings in the metastatic cervical lymph nodes were included in this study. Of these patients, 60 were male, with a mean age of 40.33 ± 13.53 years, and 98 were female, with a mean age of 43.44 ± 11.64 years.

### Comparison of clinical data, thyroid function, and primary lesion ultrasound features between the metastatic lymph node group and the nonmetastatic group

Univariate analysis revealed that compared with the nonmetastatic group, the metastatic group had a significantly greater incidence of patients aged ≤45 years, greater incidence of female sex, larger maximum diameter of the primary lesion, greater contact with the capsule, greater presence of CDFI in the primary lesion, and elevated levels of TGAb (all P < 0.05). However, no significant differences in lobulation, location, margins, shape, composition, echogenicity, internal echo homogeneity, microcalcifications, BMI, presence of Hashimoto’s thyroiditis, thyroid function laboratory markers (FT3, FT4, TSH), TPO-AB, BRAF V600E mutation, or TNM staging (all P > 0.05) were detected between the two groups (**Table 2**).

**Table 2:** Baseline clinical and US imaging characteristics of patients with PTC.

Variable	Metastatic group (n=59)	Non-metastatic group (n=99)	X²/Z value	P value
Sample Size	59	99		
Age [n (%)]				
≤45	43 (72.88)	49 (49.49)	8.313	0.004
>45	16 (27.12)	50 (50.51)		
BMI (kg/m², Mean ± SD)	25.34 ± 4.59	24.42 ± 3.56	1.156	0.248
Sex [n (%)]				
Male	30 (50.85)	30 (30.30)	6.625	0.010
Female	29 (49.15)	69 (69.70)		
Lobe Involved [n (%)]				
Isthmus	3 (5.09)	4 (4.04)	0.2	0.905
Left lobe	25 (42.37)	45 (45.45)		
Right lobe	31 (52.54)	50 (50.51)		
Location [n (%)]				
Upper third	14 (23.73)	18 (18.18)	1.075	0.584
Middle third	34 (57.63)	65 (65.66)		
Lower third	11 (18.64)	16 (16.16)		
Maximum Diameter [mm, M (IQR)]	11 (9.0)	7 (5.0)	-3.162	0.002
Contact with Capsule [n (%)]				
Yes	54 (91.53)	67 (67.68)	11.724	<0.001
No	5 (8.47)	32 (32.32)		
Composition [n (%)]				
Solid	58 (98.31)	99 (100)	1.689	0.194
Cystic-solid mixed	1 (1.69)	0 (0)		
Echogenicity [n (%)]				
Very Hypoechoic	1 (1.69)	5 (5.05)	2.211	0.331
Hypoechoic	50 (84.75)	86 (86.87)		
Mixed echogenicity	8 (13.56)	8 (8.08)		
Internal Echo Homogeneity [n (%)]				
Inhomogeneous	45 (76.27)	63 (63.64)	2.728	0.099
Homogeneous	14 (23.73)	36 (36.36)		
Shape [n (%)]				
Vertical (A/T>1)	52 (88.14)	92 (92.93)	1.052	0.305
Horizontal (A/T<1)	7 (11.86)	7 (7.07)		
Margin [n (%)]				
Irregular	50 (84.75)	85 (85.86)	0.037	0.848
Smooth	9 (15.25)	14 (14.14)		
Echogenic foci [n (%)]				
Microcalcification	49 (83.05)	80 (80.81)	0.124	0.725
Non-microcalcification	10 (16.95)	19 (19.19)		
Internal blood Flow [n (%)]				
Grade 0	20 (33.90)	10 (10.10)	14.414	<0.001
Grade I	17 (28.81)	31 (31.31)		
Grade II-III	22 (37.29)	58 (58.59)		
Hashimoto’s Thyroiditis [n (%)]				
Present	9 (15.25)	17 (17.17)	0.099	0.753
Absent	50 (84.75)	82 (82.83)		
Thyroid Function				
FT3 (pmol/L)	5.25 (0.78)	5.21 (0.86)	0.983	0.326
FT4 (pmol/L)	11.79 (3.68)	11.14 (2.42)	1.224	0.221
TSH (mIU/L)	2.02 (1.84)	2.00 (1.75)	0.634	0.526
TPO-AB [n (%)]				
Elevated	5 (8.47)	20 (20.20)	3.817	0.051
Normal	54 (91.52)	79 (79.80)		
TGAb [n (%)]				
Elevated	20 (33.90)	17 (17.17)	5.767	0.016
Normal	39 (66.10)	82 (82.83)		
**BRAF V600E mutation** [n (%)]	25.3 ± 4.6	24.4 ± 3.6	-1.156	0.248
Not tested	42 (71.19)	64 (64.65)	0.724	0.696
Yes	16 (27.12)	33 (33.33)		
No	1 (1.69)	2 (2.02)		
TNM staging [n (%)]				
Stage I	37 (62.71)	63 (63.64)	1.296	0. 523
Stage II	20 (33.90)	35 (35.35)		
Stage III	2 (3.39)	1 (1.01)		
Stage IV	0	0		

BMI, Body Mass Index; FT3, free triiodothyronine; TSH, thyroid-stimulating hormone; FT4, free thyroxine; TPO-AB, anti-thyroid peroxidase antibodies; TGAb, anti-thyroglobulin antibodies.

### Multivariate analysis of atypical ultrasound features in the metastatic lymph nodes of patients with PTC

Indicators that revealed statistically significant differences in the univariate analysis were further examined using multivariate logistic regression. The results indicated that age ≤45 years (OR = 2.898, 95% CI = 1.294–6.810), male sex (OR = 3.224, 95% CI = 1.468–7.333), contact with capsule (OR = 7.346, 95% CI = 2.448–27.049), internal blood flow (Adler grade II-III, OR = 4.915, 95% CI = 1.626–15.882), and elevated TGAb levels (OR = 5.173, 95% CI = 2.026–14.355) were identified as independent risk factors for the presentation of atypical ultrasound findings in the metastatic lymph nodes of patients with PTC (**Table 3**).

**Table 3:** Multivariate analysis of atypical ultrasound features in the metastatic lymph nodes of patients with PTC.

Variable	Regression coefficient	Standard error	Wald χ² value	P value	OR (95%CI)
**TGAb**	1.644	0.496	10.996	0.001	5.173 (2.026-14.355)
**Sex**	1.171	0.408	8.220	0.004	3.224 (1.468-7.333)
**Age**	1.064	0.421	6.388	0.011	2.898 (1.294-6.810)
Contact with Capsule	1.994	0.604	10.909	0.001	7.346 (2.448-27.049)
Internal Blood Flow Grade I	1.412	0.584	5.857	0.689	1.197 (0.492-2.893)
InternalBlood Flow Grade II-III	1.592	0.496	7.620	0.006	4.915 (1.626 - 15.882)
Maximum Diameter	0.011	0.026	0.190	0.663	0.989 (0.940 - 1.042)

TGAb, anti-thyroglobulin antibodies.

### Construction and evaluation of the nomogram prediction model

**Figure 3:**
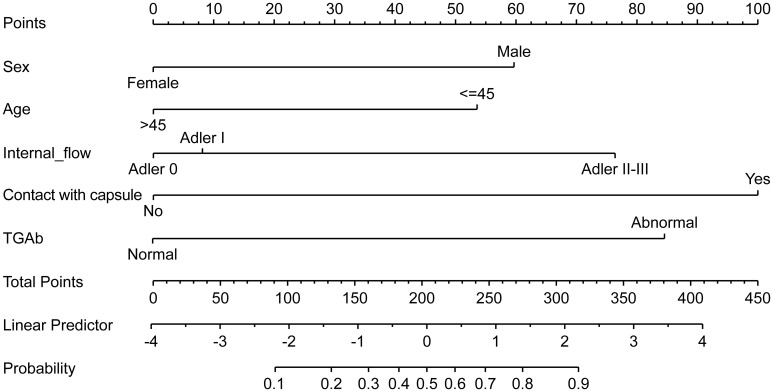
Nomogram prediction model for metastatic cervical lymph nodes with atypical ultrasound features in PTC patients.

**Figure 4:**
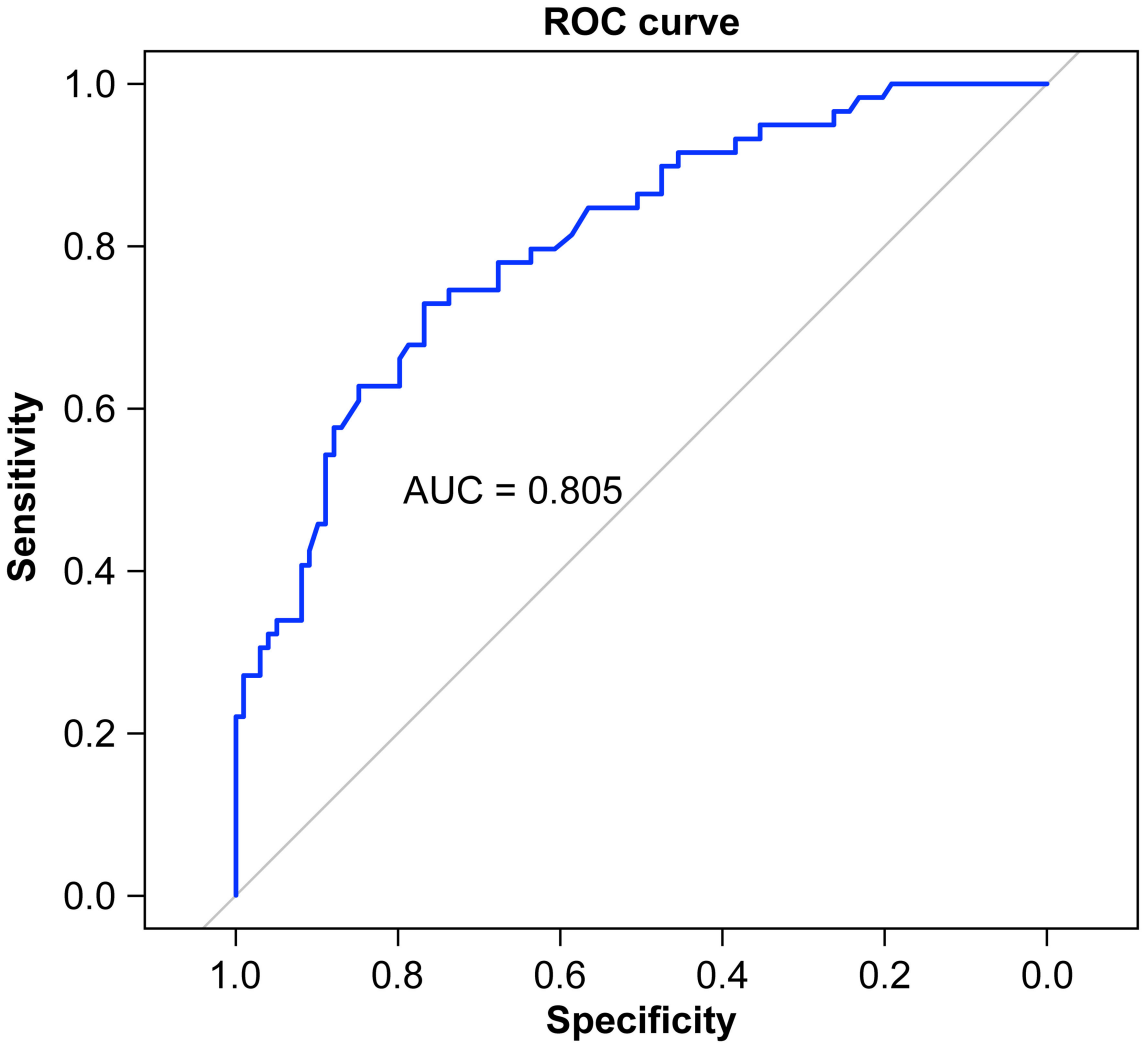
ROC curve of the nomogram prediction model.

**Figure 5:**
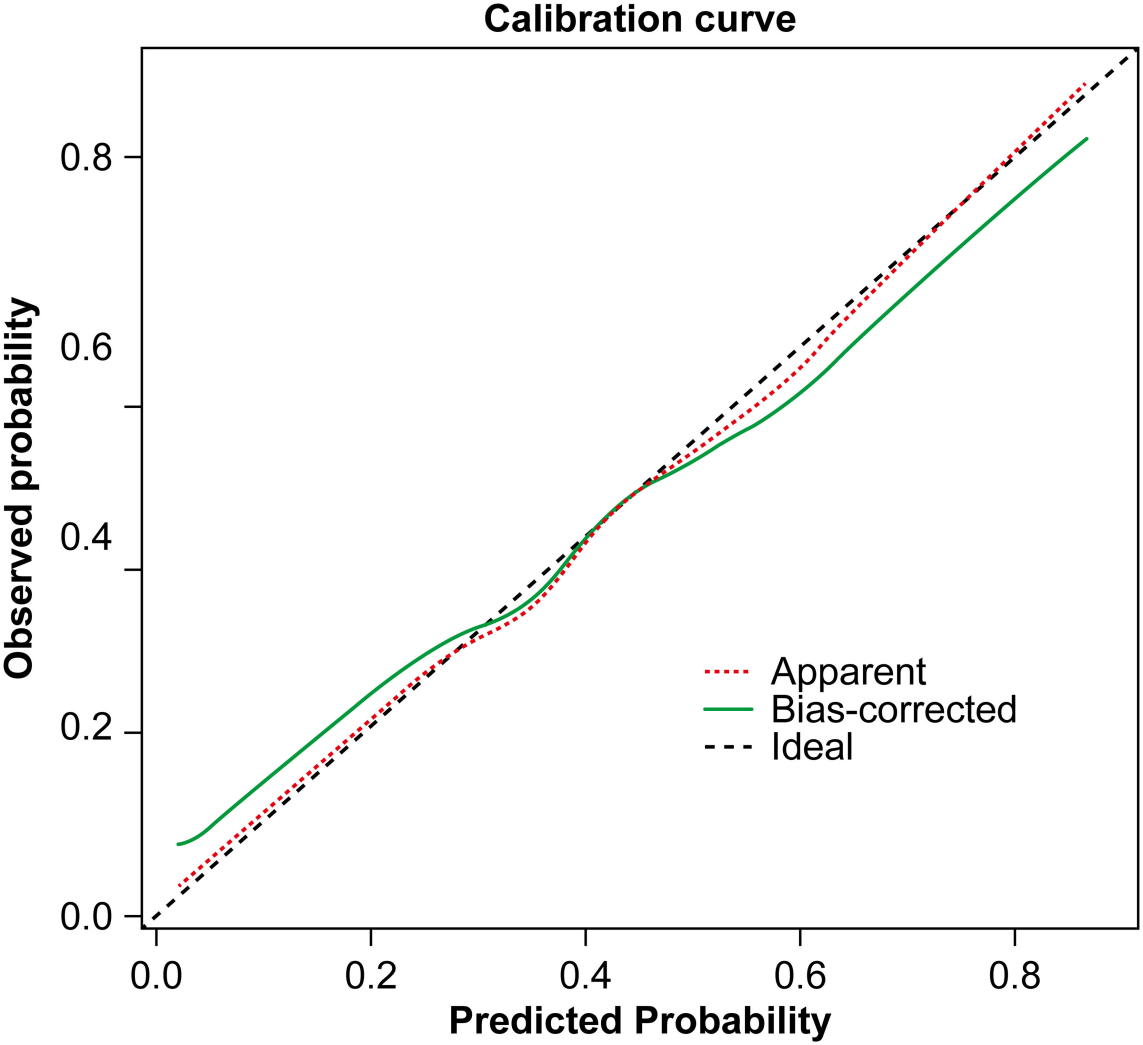
Calibration curve of the nomogram prediction model.

**Figure 6:**
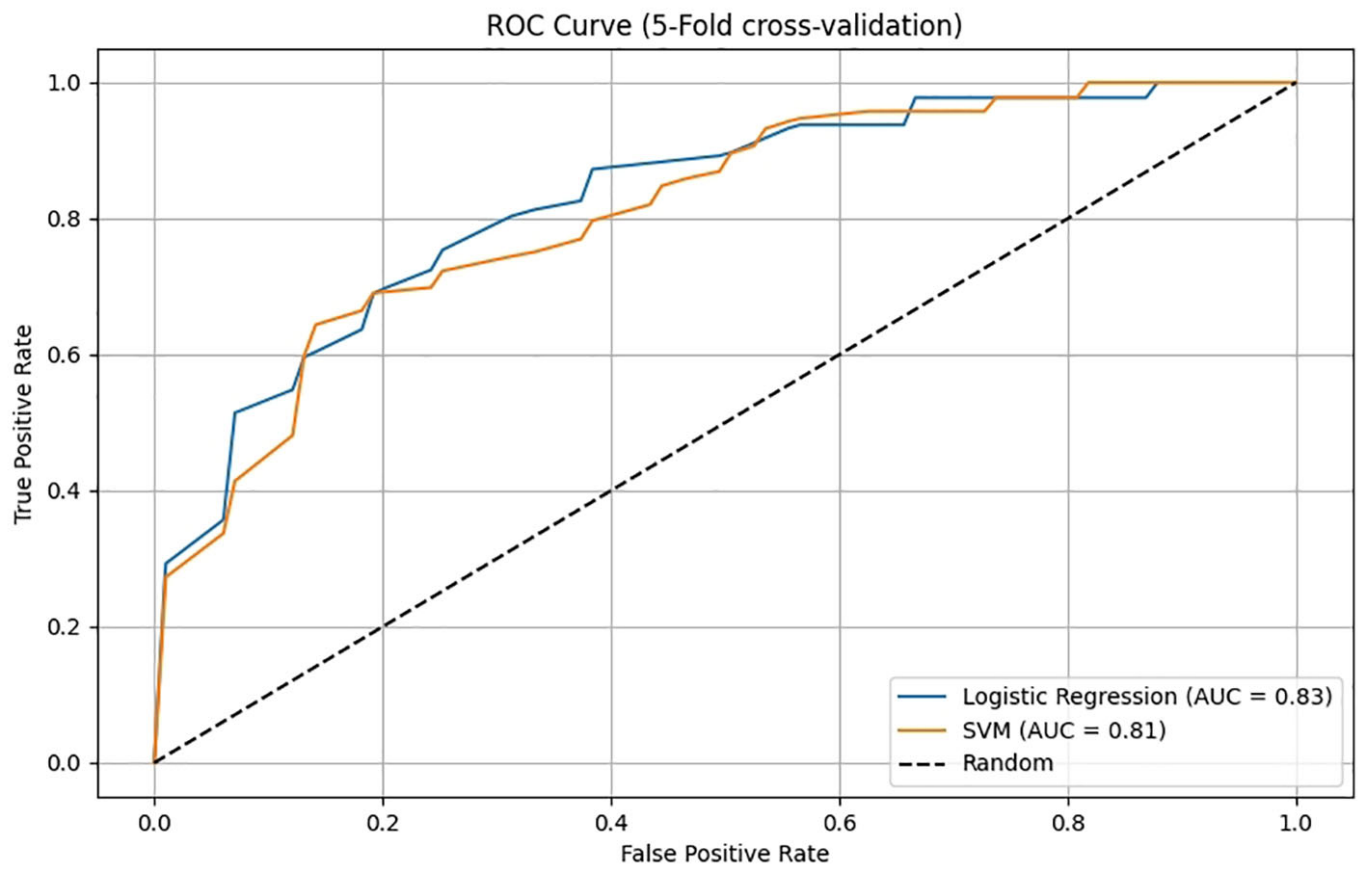
The 5-fold cross-validation of the nomogram prediction model.

A nomogram was constructed based on the results of the multivariate logistic regression analysis (**Figure 3**). The scores corresponding to each risk factor on the nomogram were summed to calculate the total score, which represents the probability of cervical lymph node metastasis in PTC patients with atypical ultrasound features. In the nomogram, males scored 60 points and females scored 0 points; 0 points for age > 45 years, 55 points for age ≤ 45 years; A blood flow distribution of grade 0 scores 0 points, a blood flow distribution of grade I scores 10 points, and a blood flow distribution of grades II-III scores 78 points; 100 points for contact with the capsule and 0 points for noncontact with capsule; normal TGAb scores 0 points and abnormal TGAb scores 86 points. The sum of the scores for the 5 risk factors is the total score, corresponding to the probability below, which is the predicted probability of developing atypical cervical lymph node metastases. The model performance was assessed using an ROC curve. The optimal cut-off value was determined to be 0.340, with an AUC of 0.805, sensitivity of 72.88%, specificity of 76.77%, and accuracy of 75.32% (**Figure 4**). The Hosmer–Lemeshow test demonstrated good model fit (R² = 10.019, P = 0.26). Furthermore, a calibration curve was generated using the Hosmer–Lemeshow contingency table to evaluate the consistency between the predicted probabilities and actual outcomes, revealing strong agreement between the predicted and observed results (**Figure 5**). The 5-fold cross-validation assesses the stability of the prediction model, and the results prove that the prediction model in this study is stable (**Figure 6**).

## Discussion

In recent years, the incidence of PTC has steadily increased (13, 14). Although the overall prognosis following surgery is generally favorable, PTC is a lymphophilic tumor with a high propensity for lymph node metastasis. Once metastasis occurs, it significantly increases the risk of local recurrence (15). Ultrasound examination of the thyroid and cervical lymph nodes is the preferred method for preoperative assessment of PTC and metastatic cervical lymph nodes (11). However, both the complex anatomy of cervical soft tissues and the fact that many metastatic lymph nodes display atypical ultrasound characteristics pose a challenge to physicians, leading to either misdiagnosis or missed diagnoses. Moreover, the accuracy of ultrasound diagnoses is often influenced by the examiner’s experience and the quality of the examination technique. Consequently, there is a pressing need to integrate ultrasound findings with the demographic characteristics and thyroid function indicators of patients to predict the risk of cervical atypical lymph node metastasis more effectively. In this study, we developed a nomogram prediction model by analyzing the ultrasound characteristics of primary PTC lesions, thyroid function laboratory markers, and patients’ clinical and pathological data. The model demonstrated promising predictive performance, with an AUC of 0.805, a sensitivity of 72.88%, a specificity of 76.77%, and an accuracy of 75.32%.

The results of this study demonstrated that pathological examination confirmed lymph node metastasis in 59 of the 158 patients with atypical ultrasound findings, which represented 37.3% of the total cohort. Both univariate and multivariate logistic regression analyses identified several independent risk factors for metastatic cervical lymph nodes with atypical ultrasound features, such as elevated TGAb levels, age ≤ 45 years, male sex, contact with the capsule, and the presence of abundant blood flow signals within the primary lesion.

Our study suggests that TGAb positivity is an independent risk factor for metastatic cervical lymph nodes in PTC patients with atypical ultrasound features. Compared with the negative TGAb group, the positive TGAb group presented a 4.2-fold increased risk of developing atypical ultrasound findings of metastatic cervical lymph nodes. Thyroglobulin is a glycoprotein secreted by thyroid follicular epithelial cells, and only trace amounts are present in the bloodstream under normal conditions. However, in cases of thyroid destruction or goiter, increased secretion into the blood makes TGAb more detectable. TGAb is an important marker of autoimmune thyroid disease (AITD) and is particularly associated with Hashimoto’s thyroiditis (also known as chronic lymphocytic thyroiditis). Its association with thyroid cancer and metastatic lymph nodes may be due to chronic inflammation promoting carcinogenesis or interfering with detection (16). Wen et al. (17) also proposed that TGAb positivity reflects an active tumor, which is typically more aggressive and more likely to metastasize. Vasileiadis et al. (18) reported that the incidence of lymph node metastasis was significantly greater in PTC patients with TGAb positivity (20.3%) than in those without (10%). A retrospective study by Zhou et al. (19) involving 2,926 PTC patients also revealed that TGAb positivity was associated with a greater likelihood of cervical LNM, further supporting our findings.

This study also revealed that, compared with patients whose primary lesions did not contact the thyroid capsule, those with tumors in direct contact with the thyroid capsule had a 6.3-fold increased risk of cervical lymph node metastasis with atypical ultrasound features. There are two potential explanations for this finding. First, a tumor in contact with the capsule may have already invaded it, which indicates a higher degree of malignancy and an increased likelihood of metastasis. Second, the thyroid’s visceral fascia and outer capsule contain rich vascular and lymphatic networks. Tumor contact with the capsule could involve blood vessels and lymphatic vessels within these layers, which significantly increases the risk of lymph node metastasis.

Generally, the incidence of tumors increases with age. However, in this study, 72.88% of patients in the atypical ultrasound metastatic lymph node group were ≤45 years, which was significantly greater than the 49.49% in the nonmetastatic group. Multivariate logistic regression analysis revealed an odds ratio (OR) of 2.9 for patients aged ≤45 years, which indicates that PTC patients under 45 years of age had a 1.9-fold increased risk of cervical LNM with atypical ultrasound features. This finding is consistent with a retrospective cohort study involving 48,166 PTC patients that reported that the incidence of lymph node metastasis decreased with age (20). Another retrospective study revealed that patients with micropapillary thyroid carcinoma aged ≤45 years were more likely to develop cervical lymph node metastasis (21). One possible explanation is that younger patients tend to have more biologically active tumor cells, which may increase their susceptibility to metastasis.

This study also revealed that the presence of CDFI in the primary lesion increases the risk of cervical LNM with atypical ultrasound features. Compared with that in the group without CDFI, the risk of metastatic lymph nodes with atypical ultrasound features in the group with an Adler grade of II-III in the primary lesion was 3.9 times greater, suggesting that rich and disordered blood flow within the PTC nodule may serve as a predictor for cervical metastatic lymph node involvement. Previous studies have indicated that as thyroid cancer progresses, tumor blood vessels are induced by angiogenic factors such as vascular endothelial growth factor (VEGF), which leads to the formation of disorganized vascular networks (22). As tumor angiogenesis increases, the interaction between active tumor cells at the margin and lymphatic vessels also increases, facilitating the metastasis of the tumor to the lymphatic system. Furthermore, another study reported that the expression of VEGF receptor 1 (VEGFR1) is a significant predictor of lymph node metastasis (23).

This study developed a nomogram prediction model for atypical ultrasound-identified metastatic lymph nodes based on five independent risk factors, with an AUC of 0.805, demonstrating good discriminatory ability. In clinical practice, this model holds significant potential. Ultrasound specialists should exercise heightened vigilance and perform thorough cervical lymph node examinations to minimize the risk of misdiagnosis for young male PTC patients with elevated TGAb levels. Surgeons can utilize this model for accurate risk stratification, enabling them to tailor surgical and postoperative treatment strategies that enhance patient outcomes.

This study has several limitations. First, this was a retrospective observational study with a relatively small sample size, which may introduce selection bias. Larger-scale studies are needed to validate our findings. Second, as a single-center study, external validation through multicenter research is needed to refine the prediction model and enhance its applicability to diverse populations. Third, previous studies have shown that the BRAF V600E mutation is associated with cervical lymph node metastasis (24, 25). This result was not obtained in this study, probably because some patients were not examined for this gene. Further research is needed in subsequent studies with larger sample sizes.

## Conclusion

In conclusion, the nomogram developed based on factors such as age, sex, contact with the capsule, blood flow signals in the primary lesion, and TGAb levels effectively predicted the risk of cervical lymph node metastasis in PTC patients with atypical ultrasound features. This model can aid in the formulation of personalized treatment plans, enhancing clinical decision-making.

## Data Availability

We are open to sharing the deidentified individual participant‐level data featured in this article upon receiving a request that outlines the study hypothesis and statistical analysis plan. Requests should be directed to the corresponding authors. The corresponding author and lead investigators will review all requests to determine the scientific merit of the proposal and decide on data‐sharing suitability. The approved applicants will be required to sign a data access agreement detailing the terms and conditions.
